# Acknowledgment of *Ad Hoc* Reviewers

**DOI:** 10.1128/mSphere.00648-18

**Published:** 2018-12-26

**Authors:** Michael J. Imperiale

**Affiliations:** a University of Michigan Medical School, Ann Arbor, Michigan, USA

## EDITORIAL

In 2018, *mSphere* continued its growth as a prime destination for researchers in the microbial sciences to publish their cutting-edge results. Our submissions continue to increase, as does the number of manuscripts that we post each week. The senior editors and editors are extremely pleased with the high quality of the work that we are seeing, and we remain committed to an author-friendly experience. As such, we value your feedback at any time. Of course, we editors cannot do our jobs without the many individuals who have served as reviewers over the past year and have provided us with critical, rapid, and fair evaluations of the manuscripts. We send our sincere thanks to all of you who have contributed your time and effort to *mSphere*.

Hector C. Aguilar
Donna Albertson
Randy A. Albrecht
Luís Manuel da Costa Almeida
Julio Alvarez
James B. Anderson
Matthew Zack Anderson
Jesse H. Arbuckle
Sergio Arce
David Armitage
Paul M. Arnaboldi
Tulika Arora
Luciana Barros Arruda
Karen Miyuki Asano
Cameron Ball
Elizabeth Ballou
Jeffrey A. Banas
Horia Leonard Banciu
Gert Bange
James D. Bangs
Stephen J. Barenkamp
Aaron M. T. Barnes
Mariana Barros
Eileen M. Barry
Naor Bar-Zeev
David Baumler
Josh Beck
Judy A. Beeler
John T. Belisle
Lindert Benedictus
Ivan Berg
Melanie B. Berkmen
Kathryn Bernard
Kyle Bibby
Derek Bickhart
Moanaro Biswas
Ibrahim Bitar
Ruth E. Blake
Steven R. Blanke
David S. Blehert
Martin Blume
Nick Bokulich
Vincent Craig Bond
Irene Bosch
Catharine M. Bosio
Elsa N. Bou Ghanem
Rebecca Brady
Curtis R. Brandt
Benjamin Brennan
Elizabeth Brickley
Jeff R. Broadbent
Christopher B. Brooke
Roland Brosch
C. Titus Brown
Harald Bruessow
Christopher Brian Buck
Bryce M. Buddle
Maria Fernanda Campagnari Bueno
Vincent Burrus
Matej Butala
Andrew P. Byrnes
Benjamin J. Callahan
Laura Campisi
Marco Candela
Rafael M. Cantón
Yongguo Cao
Paul E. Carlson, Jr.
Daniel J. J. Carr
Ronan K. Carroll
Joseph J. Carter
Maria Belen Cassera
Corinne Cassier-Chauvat
Rick Cavicchioli
Jorge L. Cervantes
Laura Cervi
Clara S. Chan
Jyotsna Chandra
Woo-Suk Chang
Narisara Chantratita
Minu Chaudhuri
Pascal Chavanet
Fei Chen
Ya-Lei Chen
James Chodosh
Chiranjit Chowdhury
Christine Clayton
Darrell Cockburn
Ian Cockburn
Aidan Coffey
Donald Cohen
Nicholas V. Coleman
Claire Colenutt
Brian Conlon
Terry D. Connell
Cynthia Nau Cornelissen
Robert A. Cramer
Paul J. Cullen
Scott Curry
Johanna Patricia Daily
Andrew J. Darwin
Jayajit Das
Raymond James Dattwyler
David J. Davido
Gargi Dayama
Harry P. de Koning
Frank R. DeLeo
Manuel Delgado-Baquerizo
Maurizio Del Poeta
Eric L. Delwart
Xiangyu Deng
Emily Derbyshire
Muriel Derrien
Jigar V. Desai
Linda C. DeVeaux
Charlene S. Dezzutti
Reshika Dhakal
Santosh Dhakal
Robert P. Dickson
Joseph P. Dillard
Kimberly Anne Dill-McFarland
Francisco Dionisio
Victor J. DiRita
Ulrich Dobrindt
Rodney M. Donlan
Kelly S. Doran
Zhicheng Dou
David J. Dowling
Charles M. Dozois
Jan Felix Drexler
Milena Dropa
Robert Duncan
Sylvia Duncan
Gary M. Dunny
Jeffrey D. Dvorin
Hideki Ebihara
Kathryn M. Edwards
Garth D. Ehrlich
Mark A. Eiteman
Nina Ekström
Vegard Eldholm
Karen L. Elkins
Kirsten Ellegaard
Ehud Elnekave
Hyungjin Eoh
Sofia Esquivel-Elizondo
Prahathees Jai Eswara
Peter Evans
Erik Falck-Pedersen
Amir Farnoud
Justin Fay
Michael J. Federle
Youjun Feng
Thomas Ferenci
Luis Fernandes
Lucia Fernandez
Scott G. Filler
Stefan Finke
Ross Fitzgerald
James M. Fleckenstein
Mike Flint
James P. Folsom
Bradley Ford
Katrina T. Forest
Jarrod R. Fortwendel
Genevieve Giny Fouda
Pilar Francino
Kristi L. Frank
Nicole Frankenberg-Dinkel
Zachary Benjamin Freedman
Hannu Fritze
Jennifer Fulcher
Iwona Gabriel
Cormac G. Gahan
James E. Galen
Michael G. Ganzle
Rozenn Gardan
Karen S. Gibb
Frank C. Gibson
Joseph James Gillespie
Maria Gomes-Solecki
Michael F. Good
Stephen V. Gordon
Patricia Grace
Andrew J. Grant
Diane E. Griffin
John F. Griffith
Charles Grose
Alexandra Gruss
Maria-Eugenia Guazzaroni
Marc-Jan Gubbels
Maria Hadjifrangiskou
Benjamin G. Hale
Scott B. Halstead
Neal D. Hammer
Sven Hammerschmidt
Xiang Y. Han
Luke D. Handke
Jason B. Harris
Freya Harrison
Roland Hatzenpichler
Kevin C. Hazen
Patrick Hearing
Robert A. Heinzen
John D. Helmann
Thomas Henry
Scott E. Hensley
Robert L. Hettich
N. Luisa Hiller
Jay Hinton
Aurélia Hiron
Sarah E. Hochman
Thomas Hoenen
Deborah A. Hogan
Martin J. Holland
Devin B. Holman
Robert Holt
Bo-Young Hong
Bruce H. Horwitz
Paul A. Hoskisson
Michael Hugler
Jiri Hulcr
Romney M. Humphries
Edward Hutchinson
Lindsey M. Hutt-Fletcher
Michael J. Imperiale
Thomas V. Inglesby
Kazuyuki Ishihara
Tim James
Anuradha Janakiraman
Ann E. Jerse
Timothy A. Johnson
Kay O. Johswich
Kristina Jonas
C. Hal Jones
Kathryn M. Jones
Matthieu Jules
Boris Julg
Esther Julian
Estelle Jumas-Bilak
Li Jun
Sheryl Justice
David Kadosh
Bjorn Kafsack
Barbara C. Kahl
Suzanne R. Kalb
Robert W. Kaminski
Osamu Kaneko
Sang-Moo Kang
Brandon F. Keele
Thomas E. Kehl-Fie
Kylene Kehn-Hall
Bart J. F. Keijser
Richard Kennedy
Robert A. Kingsley
Kimberly A. Kline
Keith P. Klugman
Jan Kluytmans
Laura J. Knoll
Sarah Knutie
Salini Konikkat
Omry Koren
Anita A. Koshy
Janne J. Koskimäki
Ákos T. Kovács
Lukasz Kozubowski
Florian Krammer
Damian J. Krysan
Katarzyna Kuczkowska
Thijs Kuiken
Sherry L. Kurtz
Maurício Lacerda Nogueira
Eric R. Lafontaine
Douglas F. Lake
Tommy Tsan-Yuk Lam
Gyanu Lamichhane
Ryan A. Langlois
Alison Laufer Halpin
Adam S. Lauring
Carlo Lazado
Helen M. Lazear
William LeBar
Francois Lebreton
James Leduc
Sangmi Lee
Katherine P. Lemon
Roger C. Levesque
Petra Anne Levin
Joseph A. Lewnard
Jianrong Li
Jean K. Lim
Xiaorong Lin
Nilton Lincopan
James Locke
Melissa Bruckner Lodoen
Olga Lomovskaya
S. Wesley Long
Torey P. Looft
Lenette Lu
Micah A. Luftig
Marina Lusic
Arthur J. Lustig
Duncan R. MacCannell
Milton Maciel
John S. Mackenzie
Michael J. Mahan
James Mahony
Burkhard Malorny
Marcos Mancilla
Thomas Manning
Antonio Marcilla
Richard Markham
Susana Martínez Flórez
Christopher J. Marx
Ruth Catherine Massey
Sébastien Matamoros
Renata Oliveira Mattos-Graner
Michael Andrew McAlear
Andrew J. McBain
Timothy R. McDermott
L. C. McDonald
Anita K. McElroy
James B. McKinlay
David N. McMurray
Alan McNally
Patrick J. McNamara
David G. Meckes
Marcela Medina
Dennis W. Metzger
Barry Milavetz
Aaron W. Miller
Chad Mire
Hari Misra
Rajeev Misra
Aaron P. Mitchell
Elena Mitsi
Tetsuya Mizutani
Claudia R. Molins
Stefan Monecke
Paul T. Monis
Cary A. Moody
Miguel A. Morales
Masamoto Morita
Yuki Morono
Donald A. Morrison
John Morrison
Lynda A. Morrison
Hunter Moseley
David M. Mosser
Mohamed Mousli
Karl Münger
Vasant Muralidharan
Timothy F. Murphy
Niculina Musat
James M. Musser
Lawrence C. Myers
Joe S. Mymryk
Indira U. Mysorekar
Lisbeth M. Naess
Sham Nambulli
Esther Ndungo
Francesca Necchi
David E. Nelson
Terry Fei Fan Ng
Kirsten Nielsen
Pablo Ivan Nikel
Peter Norberg
Joshua D. Nosanchuk
Patricia Nuttall
Austin Nuxoll
George-John E. Nychas
Andrés Obregón-Henao
Daniel O'Connor
Audrey R. Odom
Marco Rinaldo Oggioni
Anil Ojha
Antonio Oliver
Michael Olson
Teresa R. O'Meara
John J. Openshaw
Kathy Orloski
Marc Ouellette
Scot P. Ouellette
Slobodan Paessler
Malcolm G. P. Page
Raghavan U. Palaniappan
Mark John Pallen
Jonathan Palmer
Robert J. Palmer
Tracy Palmer
John C. Panepinto
Gaël Panis
Costas C. Papagiannitsis
John R. Papp
Krisztina M. Papp-Wallace
Marilyn Parsons
Marcela F. Pasetti
Joseph N. Paulson
Mattie Pawlowic
Sophie Payot
Craig Peebles
William Penwell
Susan L. Perkins
Ronald Perraut
Vincent Perreten
Renata Cristina Picão
Michael E. Pichichero
Christine Pierce
Johann Pitout
Paul J. Planet
Richard K. Plemper
Laurent Poirel
Welkin H. Pope
Chad K. Porter
Ann M. Powers
Margarita Poza
Rachel Presti
Lance B. Price
Ruben Props
Pirkko J. Pussinen
Firdausi Qadri
Udi Qimron
Frederick D. Quinn
Miguel Eduardo Quinones-Mateu
Robert G. Quivey
Sylvain Raffaele
Rahul Raghavan
Roger G. Rank
Chad A. Rappleye
J. Kamile Rasheed
David Rasko
Amit Raychoudhuri
Timothy D. Read
Christopher Rensing
Federico Rey
Todd B. Reynolds
Mark S. Riddle
Lee W. Riley
D. Ashley Robinson
Daniel D. Rockey
Katharina Roeltgen
Adela Roguet
Jeffrey A. Rollins
Sandra Romero-Steiner
David A. Rosen
Gian Maria Rossolini
Jason A. Rothman
Yoann Roumbouts
Michael W. Russell
Alexey Ruzin
Edward T. Ryan
Irene Salinas
Mauro Jose Costa Salles
Anne Salonen
Hélène Sanfaçon
Guido Santos
Abhay R. Satoskar
Mario Schelhaas
Mark A. Schembri
Jarad M. Schiffer
Patrick M. Schlievert
Patrick D. Schloss
Achim Schnaufer
Jared Schrader
Audrey N. Schuetz
Ingrid L. Scully
Christopher John Secombes
Julia A. Segre
Robert W. Shafer
Jason W. Shapiro
Rebecca Shapiro
Anukriti Sharma
Shashi K. Sharma
Gil Sharon
Thomas J. Sharpton
Alaullah Sheikh
Zhangqi Shen
Nathan M. Sherer
Paul Sheridan
Louis A. Sherman
Laura Jayne Sherrard
Daisuke Shiomi
Joshua D. Shrout
Ariel Mariano Silber
Ellen K. Silbergeld
Esther Singer
Steven M. Singer
Amit Singh
Shino Sizuki
Eric P. Skaar
Ioanna Skountzou
Younes Smani
Mark S. Smeltzer
Amber M. Smith
Paul Sniegowski
Evan Snitkin
Katharina Sobotta
Heidi Marie Soeters
Shirleen Soh
Greg A. Somerville
Nahum Sonenberg
Joseph A. Sorg
Jessica R. Spengler
Prakash Srinivasan
Henrik Stålbrand
Christopher Staley
Blake W. Stamps
Margaret Stanley
Nicola R. Stanley-Wall
Michael N. Starnbach
Jill M. Steinbach-Rankins
Sandra Steiner
O. Colin Stine
Paul D. Straight
Daniel Streblow
N. Strockbine
Jorg Stulke
David J. Sullivan
Keer Sun
Lotta-Riina Sundberg
Michael G. Surette
Yasuhiro Suzuki
W. Edward Swords
Steven M. Szczepanek
Mariusz Tadych
Stefan Taube
Stephen Taylor
Anne E. Tebo
Sam R. Telford
Vernon L. Tesh
Ken Teter
Elitza S. Theel
Kwai Lin Thong
Justin Adam Thornton
Guo-Bao Tian
Natasha L. Tilston-Lunel
Thao T. To
Marcelo Tolmasky
Eva M. Top
Lindsay Traeger
Julien Tremblay
Jenny Tung
Robert Turesky
Gregory H. Tyson
Jude E. Uzonna
Françoise Van Bambeke
Jan Roelof van der Meer
Adrianus W. M. van der Velden
Xaveer Van Ostade
Jan Peter van Pijkeren
Ophelia Venturelli
Emily Vogtmann
Uwe Volker
Veronika von Messling
H. Martin Vordermeier
Lee W. Riley
H. Walke
Jens Walter
Aiming Wang
Jianjun Wang
Ping Wang
Xin Wang
Keith Warriner
Keith E. Weaver
Friedemann Weber
Mary Weber
Matt Wecker
Brian C. Weinrick
Daniela Weiskopf
David S. Weiss
Guido Werner
Dawn Wetzel
Christopher Whitehurst
Martin Wiedmann
Patricia P. Wilkins
Candace L. Williams
Freyja Williams
Emma H. Wilson
Kevin Scott Wilson
Sam John Wilson
Fred M. Winston
Matthew C. Wolfgang
Janet M. Wood
Rachel A. F. Wozniak
Kristine M. Wylie
Yan Q. Xiong
Qingfu Xu
Chaoyang Xue
S. Steve Yan
Xiang Yang
Egija Zaura
Rui Zhang
Thomas Zhart
Hao Zheng
Baoli Zhu
Guan Zhu
Joseph M. Ziegelbauer
Luca Zoccarato
Peter Zuber
Gerardo Zuñiga-Bermudez

